# University of Buffalo

**Published:** 1856-06

**Authors:** 


*University of Buffalo.*—We have this month the pleasure of announcing that Prof. Austin Flint has accepted the Chair of "Clinical Medicine and Pathology" in the University of Buffalo, having resigned his position as professor of Theory and Practice of Medicine in the University of Louisville. In congratulating the friends of the Buffalo school, on this important addition to its strength, we feel at liberty to say that Prof. Flint's resignation at Louisville was only sent in after much deliberation, and with many regrets at the necessity of sundering a connection at once lucrative and peculiarly pleasant in its relations to his colleagues. But his strong attachment to Buffalo as a home, to the Buffalo school, of which he was one of the founders, and the disadvantages attending an absence of five months from his practice every winter, have finally decided him to remain a fixture in the city where he has so long resided.

Known to all our readers as for ten years the editor of this journal, and during that time Professor of Principles and Practice in the University of Buffalo, as the author of works on fever and on physical diagnosis, which have placed him in the first rank of American writers, and as an eloquent and highly practical lecturer, his renewed connection with the Buffalo school cannot fail to add largely to its prosperity.

It is with equal pleasure that we announce the appointment of Prof. Edward M. Moore, of Rochester, to the Chair of "Surgical Anatomy and Surgical Pathology."

Prof. Moore is widely known as a surgeon, anatomist and teacher. His style as a lecturer is ready, fluent, practical, without oratorical display, but with much grace and precision of expression. He possesses peculiar qualifications for the branches which he is now to teach. Accustomed to lecture upon anatomy, and familiar with all surgical experience, his teachings on the surgical relations of parts will bear with them the learning of an anatomist, and the practical tact of a surgeon. In surgical pathology his skill as a microscopist will add much to the interest of his chair.

Those familiar with the curriculum of the Buffalo school will recognize the fact that these appointments are *additions* to its corps of teachers, and not made to fill vacancies. Prof. Rochester continues to occupy the Chair of Principles and Practice of Medicine, in which he has already gained so brilliant and enduring a reputation. Prof. Hamilton remains as heretofore, the teacher of surgery, a department which cannot be better filled than by him. His character as a teacher and surgeon has long been a "household word" in the profession, and his recent valuable additions to the literature of fractures in his able reports to the American Medical Association, has materially added to that reputation.

The department of Physiology has now been added to that of Descriptive Anatomy. During the last two terms, the Prof. of Anatomy has been overloaded with duties, which are now much lightened by the appointment of Prof. Moore in the department of Surgical Anatomy. This will enable him to discharge the duties of his chair with greater efficiency and ease.

The usual summer announcement will soon be issued. It will show that at no previous time has the University of Buffalo possessed so strong a vitality, or so many elements of prosperity. It now has eight professors, all of them men of experience as teachers, but none of them "old fogies." It will be the effort of the faculty to provide a thorough practical course, full in all its departments, and presenting all the advantages, clinical or others, offered by any school in the country.

